# The Modular Organization of Pain Brain Networks: An fMRI Graph Analysis Informed by Intracranial EEG

**DOI:** 10.1093/texcom/tgaa088

**Published:** 2020-11-25

**Authors:** Camille Fauchon, David Meunier, Isabelle Faillenot, Florence B Pomares, Hélène Bastuji, Luis Garcia-Larrea, Roland Peyron

**Affiliations:** 1 Central Integration of Pain in Humans (NeuroPain-lab), Inserm U1028, CNRS UMR5292, Lyon Neuroscience Research Center, Bron 69500, France; 2 Aix Marseille Université, CNRS, INT (Institute of Neuroscience de la Timone), Marseille 13005 France; 3 University Jean Monnet, Saint-Étienne 42100, France; 4 Centre de Recherche de l’Institut Universitaire de Gériatrie de Montréal, Montreal, QC H3W 1W6, Canada; 5 University Claude Bernard Lyon 1, Villeurbanne 69100, France; 6 Hospices Civils de Lyon, Lyon 69002, France; 7 Service de Neurologie et Centre de la Douleur du CHU de St-Etienne, St-Etienne 42055, France

**Keywords:** acute pain, functional connectivity, graph theory, intracranial EEG, network hubs

## Abstract

Intracranial EEG (iEEG) studies have suggested that the conscious perception of pain builds up from successive contributions of brain networks in less than 1 s. However, the functional organization of cortico-subcortical connections at the multisecond time scale, and its accordance with iEEG models, remains unknown. Here, we used graph theory with modular analysis of fMRI data from 60 healthy participants experiencing noxious heat stimuli, of whom 36 also received audio stimulation. Brain connectivity during pain was organized in four modules matching those identified through iEEG, namely: 1) sensorimotor (SM), 2) medial fronto-cingulo-parietal (default mode-like), 3) posterior parietal-latero-frontal (central executive-like), and 4) amygdalo-hippocampal (limbic). Intrinsic overlaps existed between the pain and audio conditions in high-order areas, but also pain-specific higher small-worldness and connectivity within the sensorimotor module. Neocortical modules were interrelated via “connector hubs” in dorsolateral frontal, posterior parietal, and anterior insular cortices, the antero-insular connector being most predominant during pain. These findings provide a mechanistic picture of the brain networks architecture and support fractal-like similarities between the micro-and macrotemporal dynamics associated with pain. The anterior insula appears to play an essential role in information integration, possibly by determining priorities for the processing of information and subsequent entrance into other points of the brain connectome.

## Introduction

**Figure 1:**
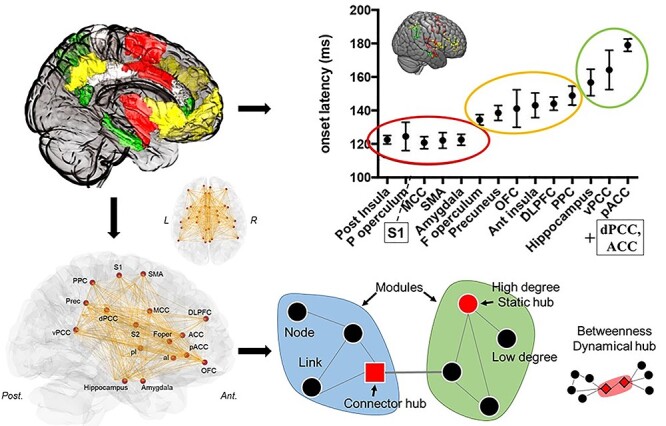
Summary of temporal dynamics of brain regions identified with stereotactic (iEEG) recordings, network construction and graph metrics during pain. Top: Onset latencies of nociceptive responses recorded with intracranial iEEG (Reproduced and edited by permission from Bastuji et al. 2016), and their corresponding anatomical localization based on the Human Connectome project atlas (see Table 1). Bottom: Graph representation of the brain network derived from functional connectivity matrix. The graph theoretical analysis and modular partition are projected onto the brain graph. Modules are represented in color (blue and green circles) where links are concentrated inside. Black dots and gray lines represent respectively nodes and links. Hubs are shown in red and categorized into three categories: static hubs (i.e., high degree: red sphere), dynamical hubs (i.e., high betweenness centrality: red diamond), and connector hubs (i.e., high between- and within-modular connectivity: red square).

The conscious perception of pain is the result of dynamic interactions of neural activities in distributed brain networks, rather than the immutable consequence of a noxious event (Tracey and Mantyh 2007; Kucyi and Davis 2015). Conscious experiences are not confined to sensory areas, but rather represented over large, interconnected portions of the cortex (Aru et al. 2012; Michel et al. 2019). It is commonly admitted that primary and secondary somatosensory areas (S1, S2) as well as the posterior operculo-insular region are involved in the discriminative processing of pain, while the mid- and anterior cingulate regions, anterior insula, prefrontal, and posterior parietal areas contribute to cognitive-evaluative processes, and limbic/paralimbic structures (temporal pole, amygdala, perigenual cortex) to affective components (Tracey and Mantyh 2007; Garcia-Larrea and Peyron 2013; Wiech 2016). Intracranial EEG (iEEG) recordings from human brain have recently characterized the activation dynamics of a number of these structures in response to phasic nociceptive input (Bastuji et al. 2016); this information has helped to develop models whereby both successive and overlapping waves of activation (see Fig. 1) sustain the transition from unconscious nociception to conscious pain (Garcia-Larrea and Bastuji 2018). These models apply, however, to the first steps of information processing that develop in less than 1 s. Although the pattern of intra- and interareal connections in the brain appears largely scale free, with “fractal” connectivity properties reproducing at short and long time scales (Nagy et al. 2017; Xue and Bogdan 2017; Racz et al. 2018), it remains unknown whether the spatiotemporal coordination described for pain perception at short temporal scales may be applied to the brain network organization at longer time periods such as those explored in fMRI-based imaging.

Graph theoretical tools can meet these demands by quantifying the local and global network interconnections and by providing a formal characterization of the brain topology, beyond what can be reached by simple functional connectivity analysis (Bullmore and Sporns 2009; Rubinov and Sporns 2010; Crossley et al. 2016). Graphs are fairly simple models, by which the brain connectome is reduced to a collection of “nodes” (brain regions), “links” (functional connectivity between regions), and “hubs” (regions with radial multiconnectivity and/or topological importance; see Glossary in Supplementary Material). The human brain, as many other complex networks, is characterized by a combination of “small-world organization” (short paths across the whole network) and modularity properties (local clustering of brain regions into strongly interconnected subsets called modules). This type of organization confers significant advantages in signal processing (Watts and Strogatz 1998; Bassett and Bullmore 2006). Decomposing brain activity into functional modules of regions to investigate their interactions, without prioritizing the connections from a seed-region, appears therefore as a well-suited approach to study pain—an experience that requires the spatiotemporal coordination of different brain regional subsets (Meunier et al. 2010).

Graph analysis of resting-state networks has recently suggested extensive reorganization of sensorimotor networks and hub topology in patients with chronic pain, as compared with controls (Mansour et al. 2016; Mano et al. 2018; Kaplan et al. 2019; Fauchon et al. 2020); the impact of these data is, however, limited by the absence of knowledge on how these structures are participating to the physiological pain experience. Thus, the main aim of this study was to characterize the functional segregation and integration of the brain network associated with the experience of pain in healthy subjects. We tested the hypothesis that the modular organization of the (sub)cortical nodes during pain can shape the temporal structure of nociceptive-related activation previously defined with iEEG (Bastuji et al. 2016). Taking as standpoint the brain areas identified by human iEEG as being involved in the transition from nociception to conscious pain (Bastuji et al. 2016), we used graph theory with modular analysis on functional magnetic resonance imaging (fMRI) recorded during noxious heat stimuli. Pain-related modular partitions were based on the weighted functional correlation matrices during pain, from two independent sets of fMRI data including 60 healthy participants, one being used for main analysis (*n* = 36) and the second for replication/validation (*n* = 24). To further assess the specificity of the network structure in pain, we compared the connectivity and graph properties to painful stimuli with those derived from auditory stimulation. Particular attention was paid to identify hub nodes, since they have a strong influence over network efficiency and facilitate information transfer among regions (Power et al. 2013; van den Heuvel and Sporns 2013).

## Materials and Methods

### Participants

A total of 60 healthy subjects (30 females, 30 males; mean age in years ±SD: 23.8 ± 4.5), all right handed, were included in the analyses. The sample included two independent groups of retrospective fMRI data, obtained at different times. Group 1 (main analysis group: *n* = 36, 18 females, mean age 25.5 ± 6.0 years) underwent the principal graph analysis, and group 2 (validation group: *n* = 24, 12 females, mean age 22 ± 2.9 years) was submitted to a replication analysis for validation of results obtained in group 1. There were no significant differences in age or sex distribution between the 2 groups (*P* > 0.4), and the methods to induce experimental pain were the same in both. All participants in both groups were screened for depression (Beck depression inventory: Beck et al. 1988) and anxiety state (State–Trait Anxiety Inventory: Spielberger et al. 1970); had no history of neurological, psychiatric, or chronic pain disease; and did not take any medication except contraceptive. All the participants provided written informed consent, and research procedures were approved by local and regional Ethics Committees (2012-A01232-41, CHU de Saint-Étienne, Comité de Protection des Personnes, Sud-Est 1, France).

**Figure 2:**
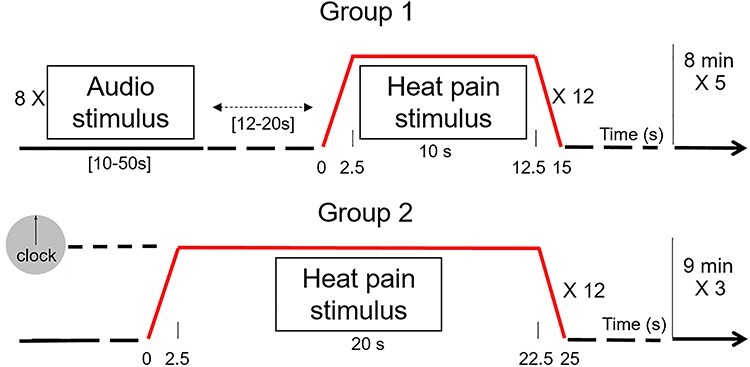
Experimental design. In both groups, participants received 12 thermal noxious stimulations in each functional session. Group 1$participants (*n* = 36) received 12 heat pain stimuli of 15 s each (including 5 s of ramps), and 8 auditory stimulations of 27.4 ± 14.3 s, separated by variable interstimulus intervals [12–20 s]. Participants from group 2 (*n* = 24, validation cohort), received 12 heat pain stimuli of 25 s each (including 5 s of ramps) no audio stim, and had their gaze fixed on a clock showing the time passing. Acute pain stimulations were fixed to a temperature inducing an intensity rated 6/10 by all the participants in both groups (see Fauchon et al. 2019).

### Stimuli and Experiments

Thermal noxious stimulations were applied on the dorsum of the left hand with a 30 × 30 mm contact probe (Pathway Pain & Sensory Evaluation System, TSA-2001, Medoc Ltd, Advanced Medical System). Nociceptive thresholds were individually determined in a pre-experimental phase before fMRI with the methods of limits. Mean pain thresholds were 46.8 ± 1.08 °C in group 1 and 44.1 ± 0.68 °C in group 2. In both groups, thermal stimuli were set to a baseline warm temperature that increased toward a painful heat temperature (i.e., rated 6/10 on a 0–10 visual rating scale) maintained for 15 s in group 1 and 25 s in group 2, including 2.5 s of ramp to reach the plateau, and 2.5 s to return at the baseline (see Fig. 2). Each functional MRI session comprised 12 noxious heat stimuli delivered at randomized interstimulus intervals (mean ISI = 23.0 ± 12.9 s). Participants in group 1 also received 8 auditory stimuli in each functional session (mean duration = 27.4 s ± 14.3; [10–50 s]). Auditory stimuli consisted of short phrases intermingled with pain stimuli (mean ISI = 51.7 s ± 33.1 s; [12–20 s]) and delivered through earphones (Nordic Neuro Lab fMRI audio system, Neuro Device, Poland; same experimental design used in Fauchon et al. 2019). Group 2 received painful stimuli exclusively, while watching a clock that indicated the stimulus duration.

### Image Acquisition and Data Pre-processing

Blood oxygen level-dependent (BOLD) fMRI signal was recorded on a 3 Tesla MR scan (Siemens, Erlangen, Germany). T1-weighted anatomical images were acquired at the beginning of the experiment with MP-RAGE sequence (voxel size 0.9 × 0.9 × 0.9).

#### fMRI Acquisition Group 1

Prisma Siemens, with 64-channels head coil: T2^*^ weighted MR signals were measured during 5 sessions of 8 min each, using an interleaved gradient echo-planar imaging (EPI) sequence (TR = 2200 ms, TE = 30 ms, voxel size = 3.0 × 3.0 × 3.0 mm, flip angle 90°, slices/volume 40). A total of 220 EPI volumes were acquired in each session.

#### fMRI Acquisition Group 2

Verio Siemens with 12-channels head coil: T2^*^ weighted MR signal were measured during three sessions of 9 min each, using an interleaved gradient echo-planar imaging (EPI) sequence (TR = 2560 ms, TE = 45 ms, voxel size = 3.0 × 3.0 × 3.0 mm, flip angle 90°, slices/volume 32). A total of 205 EPI volumes were acquired in each session.

#### Preprocessing

Identical preprocessing was applied to the 2 datasets, using Statistical Parametric Mapping software (SPM12, Welcome Trust Centre for Neuroimaging) running on MATLAB R2014b (MathWorks, Naticks). The first two functional scans from each BOLD session were deleted. Preprocessing included motion correction, co-registration of the structural and functional images and normalization with the segmentation procedure into the Montreal Neurological Institute (MNI) space. No spatial smoothing was applied before regional parcellation to avoid increasing artificially the correlation of signals in atlas-based graph functional activity (Alakörkkö et al. 2017; Gargouri et al. 2018).

### Brain Regions and Time-series Extraction for Cortical Network Analysis

Graph-based network analysis allows describing the topological properties of a network, its segregation and integration (i.e., local and global network interconnections). The patterns obtained when performing modular detection on fMRI functional connectivity networks are often quite similar to those obtained with independent component analysis (ICA; Power et al. 2011; Yeo et al. 2011). However, graph-network based approach allows for interpretation beyond the simple presence of separate modules, and provides cues on how these modules are related (e.g., through between-module connections), which is not accessible by other methods such as ICA. A brain graph has the benefit of providing a conceptually simple model without a priori, where the brain activity involved in the emergence of pain perception can be interpreted from an information-processing viewpoint (see Sporns 2018). Graph analysis was targeted to areas previously identified with iEEG as being relevant to the experience of pain. It was therefore based on a network of brain regions consistently activated during the first second following a noxious thermal stimulus, and whose temporal and spatial dynamics could be described using iEEG recordings (Frot et al. 2008, 2014; Bastuji et al. 2016). Thirty-four regions (17 per hemisphere) were considered, including insular, posterior and anterior parietal, prefrontal, cingulate, hippocampal and limbic (amygdala) areas (see Fig. 1 and Table 1). Regions of interest (ROIs) were defined on the basis of the position of electrode contacts used to determine EEG activations (Bastuji et al. 2016), and corresponding to the HCP atlas parcellation of the brain (Glasser et al. 2016). All the brain structures included in the analysis have been shown to be consistently activated by thermal pain stimuli (Apkarian et al. 2005; Tracey and Mantyh 2007; Garcia-Larrea and Peyron 2013; Wager et al. 2013; Fauchon et al. 2019).

**Table 1:** Brain regions of interest (ROIs or nodes) considered for brain network analysis

ROI (node) name	HCP atlas area name	MNI coordinates (*x*, *y*, *z*)	
		Right	Left
Parietal operculum (S2)	OP1–4	(52, −15, 15)	(−51, −17, 15)
Posterior insula (pI)	Ig, Pol1, Pol2	(39, −9, 1)	(−39, −9, 0)
Anterior insula (aI)	AAIC, AVI, MI	(36, 15, −4)	(−35, 14, −4)
Frontal operculum (Foper)	FOP1–5	(40, 11, 6)	(−40, 9, 6)
Primary somatosensory cortex (S1)	1, 2, 3a–b	(41, −25, 51)	(−41, −26, 51)
Supplementary motor area (SMA)	SCEF, 6ma, 6mp	(14, 0, 63)	(−11, −1, 63)
Perigenual ant. cingulate cortex (pACC)	a24	(3, 39, 0)	(−4, 40, 0)
Anterior cingulate cortex (ACC)	p24	(2, 36, 16)	(−3, 36, 16)
Mid-cingulate cortex (MCC)	a24pr, p24pr, 33pr	(3, 7, 36)	(−3, 10, 34)
Dorsal post. cingulate cortex (dPCC)	23d, d23ab	(1, −29, 35)	(−3, −31, 34)
Ventral post. cingulate cortex (vPCC)	v23ab	(3, −53, 18)	(−4, −55, 17)
Post. parietal cortex (PPC)	PG(p, s, i), PF(m, t, op), IP0–2	(50, −44, 42)	(−49, −47, 41)
Precuneus (Prec)	7m, 31a, 31pv, DVT, 31pd	(7, −46, 39)	(−7, −44, 39)
Orbito frontal cortex (OFC)	10d, 10v, 10pp	(6, 54, −6)	(−7, 57, −4)
Middle frontal gyrus (DLPFC)	8c, 9a, 46, 9-46v/d	(34, 42, 24)	(−35, 40, 24)
Amygdala	amyg	(22, −3, −21)	(−24, −6, −21)
Hippocampus	EC, H, PeEc	(24, −18, −21)	(−24, −18, −22)
*Primary auditory cortex (A1/Heschl)*	*A1, (P, M, L)Belt*	*(50, −19, 7)*	*(−48, −21, 5)*

Notes: The nodes used in the analyses were defined from iEEG electrodes localization (Bastuji et al. 2016), and the corresponding areas in the HCP atlas (MMP 1.0, Glasser et al. 2016). These nodes focus on areas for which we know the temporal dynamics in response to nociceptive input. The right and left MNI coordinates of the 17 nodes are reported.

### Network Construction: Pain Stimulation-based Correlation Matrices

Graph analysis was performed under the graphpype functions of Neuropycon package (Meunier et al. 2020; https://neuropycon.github.io/graphpype/.) using the open-source Python package “nipype” (Gorgolewski et al. 2011). Raw time-series averaged over the voxels within each ROI were extracted for each subject. Head movement parameters, as well as average signals in the white matter and CSF were regressed out from ROI time series, and residuals were high-pass filtered (>0.01 Hz) to remove the scanner drift component of the signal and then normalized into *Z*-score for each session. Functional connectivity (FC) was derived from time series, to describe patterns of statistical dependence among neural elements (Smith 2012).

To compute stimulation-based correlation matrices, we used the methods described by Meunier et al. (2014). The regressors corresponding to the full duration of experimental stimuli were considered and convolved with a canonical hemodynamic response function (HRF). Only the positive and null parts of the convolved pain regressor (i.e., modeling the onset, end and duration of the heat noxious stimulation) were used to compute weighted correlations on pain stimulation. This aims at giving more influence on periods during which the regressor (i.e., the pain stimulus) was high, and lower influence when the regressor was low or null. For each subject, we subsequently generated a matrix of FC by computing Pearson’s correlations between the weighted time-courses of every pair of nodes, which resulted in a set of 34 × 34 symmetric pain-based correlation matrices. The correlation values were converted to *Z*-scores using Fisher’s *Z*-transform for subsequent graph analysis (Dodel et al. 2005).

**Figure 3:**
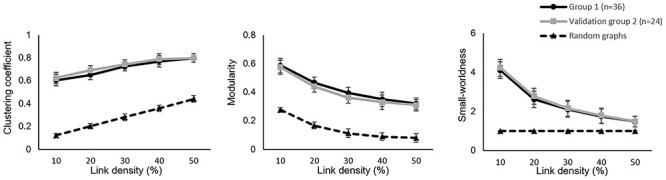
Global graph properties. The two groups of healthy participants, group 1 (*n* = 36, black lines) and group 2 (validation *n* = 24, gray lines) showed similar clustering coefficient, modularity and small-worldness coefficient across the link density thresholds tested, but significantly higher (*P* < 0.001) than equivalent random networks (black dotted lines). Data plotted as mean ± SD.

A network density threshold was applied on each individual correlation matrix to ensure the distributed nature of the brain network (“network sparsity”) and to remove weak correlations (i.e., reduce noise and variability between each connectivity matrices; Power et al. 2010). The threshold level influences network properties: increasing the density threshold enhances the number of links in the network and reduces the number of modules. Hence, applying very low threshold values will produce a disconnected network while very high values will produce highly connected network. In order to show the reliability of the results, the density threshold was applied over a range of values beginning with connections in the top 10–50% (see Meunier et al. 2009). This range of thresholds was chosen to focus on sparse but fully connected network with nonrandom aspects, rising until the network, became more densely connected. The average *Z*-correlation matrix was created by averaging the connectivity matrices of all the participants after thresholding to characterize the average modular structure during pain. The graph illustrations presented are for 30% link density threshold, since all nodes are included in the giant connected component, and graph metrics (e.g., modularity) are stabilized (see Fig. 3 and Results section). Visualization of anatomical nodes on a brain surface was created using the open-source python software Visbrain (Combrisson et al. 2019).

### Network Topological Features

The computation of graph metrics was mostly based on “Radatools” software, a set of freely distributed applications to analyze complex networks (http://deim.urv.cat/~sergio.gomez/radatools.php). The following common global network properties were computed at various link densities individually (Rubinov and Sporns 2010): 1) clustering coefficient (C), a measure of the degree to which nodes in a graph tend to cluster together; 2) shortest path length (L), defined as the average minimal number of links needed to reach any node from each node; 3) modularity (Q), defined as a set of nodes that have many intramodular connections but sparser intermodular connections, indicating a decomposability of the system into smaller subsystems (Bullmore and Bassett 2011); and 4) assortativity (A), or the correlation coefficient between the degree of a node and the average degree of its neighbors. We also computed the small-worldness coefficient, which uses a ratio of network clustering and path length compared to its random network equivalent [(*C*/*C*_random_)/(*L*/*L*_random_)]. Graph network have “small-world” properties if ratio >1 (Telesford et al. 2011). Small-world networks show more clustering coefficient than a random graph but maintain a similar shortest path length (Watts and Strogatz 1998). Though some of these graph metrics may represent some common information, each property contributes unique information to the picture of organization during pain. Also see Glossary of graph terms in Supplementary Material.

### Weighted Signed Modularity and Hub Region Detection

Modular decomposition of a graph aims at determining subgraphs (i.e., modules or communities) whose nodes interact significantly more strongly together than they do with nodes belonging to other modules (Fortunato 2010). Several algorithms have been proposed to detect the modular partition (Newman 2006), and here we used the one described by Gómez et al. (2009), implemented in Radatools software, which enables the analysis of modular structure in weighted signed graphs. Radatools is one of the few tools to offer modular partition on weighted signed networks; it was previously applied in neuroimaging studies (Meunier et al. 2014). The modularity detection was optimized across 100 iterations using the sequencing method “tfrf” (i.e., “tabu search,” “fast algorithm,” “repository algorithm” and “fast algorithm”).

Identifying brain regions that play an important or topologically central role in a network provides insight about where and how information is mediated between brain networks. Hub identification have also important clinical implications (Fornito et al. 2015), since they are known to be more vulnerable in brain disorders including chronic pain (Mansour et al. 2016). Hubs identification during the induction of pain was based on four graph metrics (see Fig. 1 and Glossary in Supplementary Material): the degree centrality, the betweenness centrality, the within-module degree, and the participation coefficient. Degree centrality is defined as the sum of links per node. Betweeness centrality of a node is defined as the density of minimal paths between two other nodes that pass through it (i.e., the most direct route between two nodes in the network). Nodes with high betweenness centrality, lie on many shortest paths mediating a high proportion of information flow, and thus represent central elements in establishing efficient communication in the network structure (Joyce et al. 2010). Nodes with a value of degree and betweenness centrality significantly higher than a random network (*P* < 0.01) were respectively categorized as static hubs and dynamical hubs. To investigate the nodal roles in intra- and intermodular communications, we measured the within-module degree (WMD) and the participation coefficient (PC) (Guimerà and Nunes Amaral 2005; Guimera et al. 2007). The WMD measures the number of links of a node compared to other nodes of the same module, whereas the PC quantifies whether a node is extensively linked to all other modules or not in the network. Nodes with WMD > 1.0 and high PC > 0.45 (i.e., mean PC + 1 SD) were defined as “connector hubs” (Meunier et al. 2009). Connector hubs link different modules together, and thus play a central role in the network organization during pain.

The significance of network topological properties (i.e., global and nodal graph metrics and hub status) during pain was assessed by comparing them to random networks with same size and number of links, using the same analysis pipeline and permutation tests. The values in the average connectivity matrix were shuffled 5000 times, and then computing same pipeline on the shuffled matrix (thresholding, network properties, modular decomposition, etc.). This test is preferred because of the expected non-normal distribution of differences in network measures. False discovery rate (FDR) correction was applied (Fornito et al. 2016).

### Comparison with the Network Structure Derived From an Audio Control Condition

Group 1 (*n* = 36) also received auditory stimuli (*n* = 8 in each functional session; Fig. 2) separated in time from pain stimuli to allow comparison of activity (see Fauchon et al. 2019). We assessed the specificity of the pain modular network by comparing it to the modular structure derived from neural activity associated with auditory stimulations. In a previous study (Fauchon et al. 2019), we showed that these 2 sensory modalities recruited distinct and common cortical and subcortical areas. Introducing responses to auditory stimuli was a means of investigating processing commonalities between two modes of stimuli deeply dissimilar, hence contributing to understand which networks and nodes were linked to the processes leading to a perceptual experience, irrespective of the sensory origin of the stimulus. Thus, the network structure in audio was used as a control condition to compare the distribution of connectivity and network properties in pain. We also added the primary auditory cortex (i.e., A1 including Heschl area, see Table 1) to the previously analyzed network (resulting of 18 nodes in each hemisphere), as a control brain region specifically associated with an audio modality. Using the same methodology and pipeline of analysis, audio and pain stimulation-based correlation matrices 36 × 36 were computed and thresholded using identical procedures as described above.

Significant condition differences on functional connectivity and graph properties between pain and audio, were evaluated using permutations tests by shuffling 5000 times and comparing the original difference between conditions to the distribution of differences after permutation. FDR correction was applied to avoid false results due to pure chance.

### Validation Analysis

The same procedure and analysis pipeline described above was also applied separately to the second group (*n* = 24 subjects), recorded during a separate session and considered here as a validation cohort. The experimental pain stimuli were longer (i.e., 25 s) than in group 1, but was calibrated to induce the same pain intensity rated 6/10 by the participant (Fig. 2). Data were not recorded in the exact same technical conditions (i.e., type of scanner, headcoils, number and duration of functional sessions), but these external differences participated to the generalizability of the findings. Pain stimulation-based correlation matrices were created and weighted signed modular analysis was applied an identical procedure as described above for group 1, in an attempt to replicate results obtained with the first set of data.

## Results

### Topological Properties Induced by Acute Nociceptive Inputs

The functional graph network associated with acute pain stimulation was consistently modular over the entire range of density thresholds (Fig. 3, 10–50%), meaning that the brain regions could be reliably segregated into strongly interconnected modules. These graph networks showed significantly larger modularity (M (mean at 30% link density ± SD) = 0.40 ± 0.04) than equivalent random graphs, thus supporting the existence of a highly modular structure (*P* < 0.001). Typical features of small-world organization were found in the structure of pain-related networks, which were characterized by higher mean clustering coefficient (*C* = 0.75 ± 0.1, *P* < 0.001) and assortativity value (*A* = 0.55 ± 0.2, *P* < 0.001) relative to random networks, but with similar shortest absolute path length (~1.2). These features suggest that the activity of brain regions during pain processing are more locally clustered compared to random networks, and have short path lengths linking all nodes even though most nodes are not neighbors of one another (small-worldness value = 2.16 ± 0.4). As these measures did not present major changes across the density thresholds tested, and stabilized at 30%, such value was chosen as representative for the subsequent results (also see Supplementary Table 1).

### Human Functional Pain Networks are Modular

**Figure 4:**
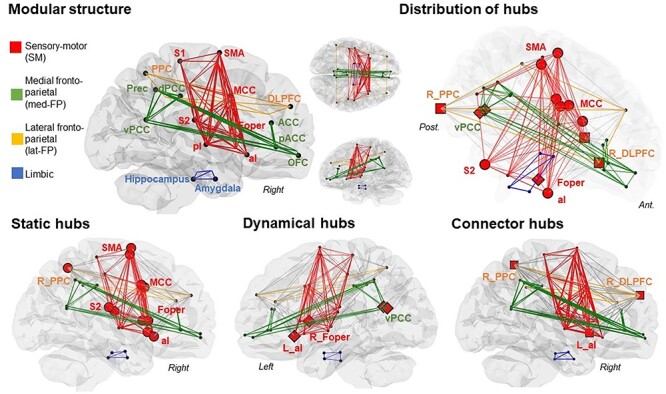
Modular structure of functional networks in acute pain stimulation. Top: anatomical representation of average population networks weighted on pain stimuli. Each module and its intermodular connections are associated with one color: sensory-motor (red), medial fronto-parietal (green), lateral fronto-parietal (orange), limbic (blue). Gray edges represent intermodular connections and red dots represent the hub regions. Bottom: anatomical representation of significant hubs in each module as compared to random networks. Static hubs correspond to regions in the network with high centrality degree (red spheres), dynamical hubs are regions with high betweenness (red diamonds), and connector hubs are regions with high within-modular degree and participation coefficient (red square); (*P* < 0.05, false-positive correction).

The modular organization of our network during pain was made up of four modules, which varied in size from 4 to 14 nodes (depicted in Fig. 4). Each module is made of a subset of brain regions that are strongly interconnected with each other and sparsely interconnected with regions in other modules. No significant interhemispheric differences were found. The largest module (14 nodes) included S1, S2, posterior and anterior insulae (pI and aI), frontal operculum (Foper), supplementary motor area (SMA), and mid cingulate cortex (MCC). These areas are all related to a sensory-motor network (SM), except for the anterior Insula (aI), which is active across multiple task domains and showed here a large number of connections within the network. The second largest module (12 nodes) included anterior and posterior midline regions: precuneus (Prec), ventral and dorsal posterior cingulate cortices (vPCC and dPCC), anterior and perigenual cingulate cortices (ACC and pACC) and medial orbito frontal cortex (OFC), and was labeled medial fronto-parietal module (med-FP). A third module (4 nodes), was labeled lateral fronto-parietal (lat-FP) module, since it mainly included posterior parietal (PPC) and dorso-lateral prefrontal (DLPFC) areas. The last module (4 nodes) included the amygdala and the hippocampus and was labeled limbic module. Overall, the 4 modules were highly intraconnected (from 58–100% intramodular mean coefficient), and also well interconnected. The SM module was largely connected with the lateral FP module (25 interlinks), whereas the medial FP module showed few links (4 interlinks) with the SM and lateral FP modules. The limbic module had lower intermodular connections with the other modules across the network (i.e., no intermodular links at 30% density threshold).

### Node Roles and Hubs Detection During Painful Heat

To investigate the local topological structure, we assigned hub status to nodes by taking into account the quantity and quality of connections in the modular network. Assessment of the connectivity profile of each node during pain compared to random networks informed that hub regions were broadly distributed across the modules (Fig. 4). We defined three categories of hub: static (high degree), dynamical (high betwenness centrality), and connector hub (high participation coefficient). The SM module had numerous inter- and intramodular connections, 9 out of 14 of its constitutive nodes had a high-degree centrality (i.e., an important number of links) and were categorized as static hubs (i.e., bilateral Foper, aI, MCC, SMA and left S2; Fig. 4). Thus, the SM module was highly integrated in the network organization during pain. Furthermore, the left aI and the right Foper had also a high betweenness centrality (i.e., shortest path), and were classified as dynamical hubs putatively playing a key role in establishing efficient communication in the network. This was specially the case of the left anterior insula, which had also a stronger within-module degree (WMD) and participation coefficient (PC) compared to other nodes. It connected 92% of the nodes within its module and also had a large number of intermodular connections with the 2 fronto-parietal modules (i.e., DLPFC, PPC, vPCC, pACC, ACC). It was therefore identified as a “connector hub,” with the potentiality to provide connections between the SM module and other modules. The three other modules were also highly intraconnected. A second structure of integration was identified in the bilateral vPCC in the medial FP module and was classified as dynamical hub; whereas the lateral FP module had two important nodes connecting the other modules, the right PPC and right DLPFC, which were identified as connector hubs (i.e., high WMD and PC; Fig. 4). None of the nodes in the limbic module could be identified as a hub (see Supplementary Table 2 for nodal graph values).

### Distinct Network Topological Features between Pain and Audio Stimulation

**Figure 5:**
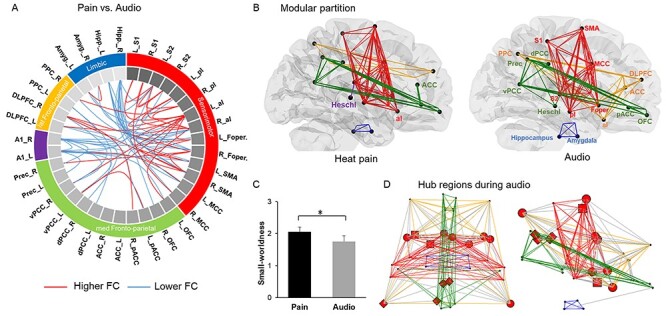
Differences in graph connectivity pattern between pain and audio stimuli. (*A*) Differences in functional connectivity (FC) between conditions, whereby the lines indicate higher (red) and lower (blue) FC during noxious heat versus audio stimulation. Colors in the outer ring show the modules with their corresponding brain regions. (*B*) Modular partition weighted on thermal noxious stimuli, and audio stimuli (at 30% link density). Auditory cortex (A1 or Heschl) nodes were fully integrated in one module, creating several connections during audio, but were isolated in pain. (*C*) Small worldness was significantly lower in the audio condition (SM = 1.76 ± 0.16) compared to pain (SM = 2.06 ± 0.17). (*D*) Distribution of hubs was widespread in the network associated with audio stimulation. Static hubs (red spheres) were represented based on the degree, dynamical hubs were defined on the betweenness centrality (red diamonds: dPCC, left: aI, PPC, Prec.) and connector hubs were defined according to their values of within-module degree and participation coefficient (red square: left aI and left SMA). ^*^P < 0.01, FDR corrected.

Significant functional connectivity differences between pain and audio stimulation are depicted in Figure 5. Comparison of pairwise correlations showed that almost half of the nodes significantly changed the strength of their FC between the 2 conditions (*P* < 0.01, FDR corrected). For the aim of this comparison, we added as a control region, the primary auditory cortex (A1), which is commonly activated by audio stimuli. The interhemispheric correlation of A1 nodes was significantly higher (*P* = 0.001) in audio stimulation (*r* = 0.85) than in pain stimulation (*r* = 0.63). Conversely, during pain stimulation, several pairs of connections were significantly stronger than in the audio condition, including mainly nodes within the sensorimotor module (e.g., S1, S2, SMA, pI, aI, Foper). For instance, the correlation strength between the posterior insula and the anterior insula was significantly stronger (*P* = 0.006) in pain (*r* = 0.72) than in audio (*r* = 0.52). Higher FC were also found between the SM module and nodes of the FP modules (i.e., vPCC, dPCC, DLPFC). Most of the connections of A1 were lower in pain, including the ones with the limbic module (i.e., hippocampus). The network average strength (i.e., the sum of connectivity values after thresholding) was significantly higher during pain compared with audio stimulation (*P* < 0.001).

### Network Topological Features Changes in Pain versus Audio

Pain was associated with lower average path length (*P* = 0.004), and higher small worldness (*P* < 0.01, FDR corrected see Fig. 5*C*). The modularity was slightly higher (*P* = 0.041) during pain (*M* = 0.43 ± 0.03) than audio stimulation (*M* = 0.35 ± 0.04), highlighting a different functional organization of nodes’ connections in the network between the 2 conditions. Conversely, neither the assortativity value nor the clustering coefficient showed significant differences between the two conditions.

The changes of connectivity in the network led to a different pattern of modular segregation during audio stimulation, which gave rise to four distinct and connected modules (spatial distribution is depicted in Fig. 5*B*). The anterior insula, Heschl, and anterior cingulate cortex were segregated in different modules than in the pain condition.

The proportion of links between- and within modules was different between pain and audio. Lower intermodular connections (*P* = 0.02), and higher intramodular connections (*P* = 0.003) were present in the pain condition relative to audio stimulation. Compared to the audio condition, the within-module degree (i.e., proportion of links per module) was higher in S2, pACC, vPCC, amygdala and lower in A1 and left SMA in pain; the participation coefficient was higher in left vPCC, and right DLPFC and lower in A1, SMA, dPCC, and MCC in pain (*P* < 0.01).

The distribution of hubs was also different between the pain and audio conditions, as illustrated in Figure 5*D*. During audio stimulation, S2 had fewer links than in pain condition and was not classified as a static hub. The left aI was identified as dynamical and connector hubs in both conditions, but in contrast during audio we found that the dPCC, left PPC, and Precuneus were dynamical hubs and the left SMA was a connector hub relative to random networks (*P* < 0.01).

Thus, a pain stimulation enhanced the modularity and small worldness in the present brain network compared to audio stimuli. The connectivity of some nodes were stimulus-dependent, for instance some of them were highly connected under pain condition (e.g., nodes within the SM module), but other nodes were only connected under audio stimulation (e.g., A1), which provides evidence for pain-dependent changes in network organization and communication. The integration was more internal with a higher proportion of connections within the modules, and pain-specific connector hubs dispatched the information between the modules as compared to audio stimulation.

### Replicability of Results in the Validation Group

When applying the same analysis pipeline on the validation cohort (group 2, *n* = 24), the above-mentioned findings on the brain network organization during pain (i.e., intra- and intermodule connectivity) remained identical. The network structure had small-world properties (see Fig. 2) across all density thresholds tested with a distribution into four identical modules as group 1 (Fig. 4), and significantly different from random networks (*P* < 0.001). The hubs were also almost identical, with the sole exception of the right Foper, which was not classified as a hub because a lower betwenness centrality. As was the case in group 1, the left anterior insula appeared as a preponderant hub in the network organization of group 2, showing a high degree and betweenness centrality, as well as a higher WMD and PC than other nodes. This region could therefore be classified the main connector hub, linking the modules with each other in the network (see Supplementary Table 2 for nodal graph values).

## Discussion

The present results suggest that the brain regions activated during a pain experience are organized in four consistent modules, a robust pattern that could be replicated in a second independent sample of subjects. Such topological aspects appear relevant to shape the consecutive waves of nociceptive-related activation previously defined with iEEG (Bastuji et al. 2016; Fig. 1). Their similarities and differences are discussed in what follows.

### A Sensory-Motor-Insular Module

The regions included in the sensory-motor (SM) module overlap with the network activated with shortest latencies following a noxious stimulus (Lenz et al. 1998; Frot et al. 2008; Ohara et al. 2008; Bastuji et al. 2016), which congregates direct cortical targets of the spinothalamic system (Dum et al. 2009). This module may therefore correspond to a first surge of activation (Fig. 1) associated to the reception of ascending nociceptive input and its preconscious encoding in sensory and motor-premotor orienting areas.

This module also included the anterior insula (aI), which is not a direct spinothalamic recipient and functionally differs from posterior insular sections and sensory-motor areas (Wiech et al. 2014). While a modular segregation is often taken as a sign of functional coupling, this should not be taken here as the processing of a selective aspect of pain. Like “Russian dolls,” each module of connections can be further partitioned into a set of functional submodules (Meunier et al. 2010). In the SM module, three functional “units” can be dissociated based on the previous literature (Tracey and Mantyh 2007; Garcia-Larrea and Peyron 2013): a “sensory” unit involves the posterior operculo-insular cortex, S2 and S1 supporting sensory-discriminative aspects of location, quality, and intensity of the stimulation (Segerdahl et al. 2015; Garcia-Larrea and Mauguière 2018). The MCC/SMA constitute a “premotor” unit that supports rapid, largely preconscious preparation of motor response (Frot et al. 2008). Finally, a “salience detection” unit involving the anterior insula, and possibly the frontal operculum, appears relevant to the detection of behavioral relevance of stimuli, not exclusive to pain. Mathematical clustering of these three subsystems in one module may be explained by their heavy anatomo-functional interconnections (Augustine 1996; Bastuji et al. 2018; Nomi et al. 2018), and by their being all activated almost simultaneously by nociceptive input (less than 80 ms separate the activation from posterior and anterior insulae; Isnard et al. 2011; Frot et al. 2014). This module appears therefore to integrate sensory encoding with stimulus salience and motor control. The sensory components of the module may reflect nociceptive-specific activity, as indicated by stimulation and lesion studies in humans (Garcia-Larrea et al. 2010; Segerdahl et al. 2015; Garcia-Larrea and Mauguière 2018); conversely, its motor, premotor, and antero-insular components appear as nonpain-specific contributions that can be recruited by any “salient” sensory input (Mouraux et al. 2011), including audio stimuli in this study (Fig. 5). Activity and connectivity within and between this module were, however, significantly higher for pain than for audio stimuli, and may participate to inform higher order networks on the physical-somatic nature of the stimulus. Indeed, the “neurological pain signature” predicting physical acute pain and described by Wager et al. (2013) largely overlaps with the SM module. It was recently shown that an identical “neurological pain signature” has the potential to drive widely different subjective pain perceptions across different ethnic groups (Losin et al. 2020). This underscores that the pain experience cannot be reduced to such initial sensory-motor-salience networks, but crucially needs interactive activity at different brain levels, here described as communicating functional modules. Accordingly, the SM module was intensively connected with other modules, notably through the anterior insula, suggesting that it may be not only involved in the initial encoding of nociceptive information, but also likely to receive “top-down” feedback from higher order regions (Dehaene and Naccache 2001).

### Associative and Multimodal Modules

The second and third modules involved brain regions of higher order in the hierarchy of noxious stimulus processing, i.e., associative and multimodal areas with longer response latencies than the regions described above (Fig. 1). The “lateral fronto-parietal” module, anchored in DLPFC and lateral PPC, is commonly associated with the central executive network (CEN; Seeley et al. 2007). This network contributes to many neurocognitive functions including decision-making and working memory (Collette and Van der Linden 2002) and is crucial for the attentional system (Norman and Shallice 1986). A strong functional connectivity between regions of CE, salience, and sensory networks has been considered as a critical step to ensure access to consciousness of sensory stimuli, both innocuous and nociceptive (Boly et al. 2007; Garcia-Larrea and Bastuji 2018).

The “medial fronto-parietal” module comprised anterior and posterior midline regions, including those forming the core of the “Default Mode Network” (DMN; (Fox and Raichle 2007). In the same vein as the SM module, distinct functional units can be dissociated here. Activation of the postero-medial cortex (posterior cingulate (PCC) and Precuneus) appears central to self-consciousness (Demertzi et al. 2013), and has been involved in pain modulation by the comments that others express on our behavior (Fauchon et al. 2019). On the other hand, the perigenual and orbitofrontal responses of the DMN have been interpreted as a link between pain awareness and descending pain-control pathways. Indeed, co-activation of these areas and modulatory brainstem regions such as the periaqueductal gray has been shown in pain adaptation mechanisms in healthy subjects (Coulombe et al. 2016), and abnormalities of this circuitry are also reported in chronic pain conditions (Ren and Dubner 2002). In iEEG recordings, activity in this module persists after the stimulus has produced an overt motor or verbal response (Bastuji et al. 2016), suggesting a role in both the consolidation of immediate perceptions, and in the control of adequate behavioral reactions.

### Amygdala and Hippocampus

The limbic module, made up of the amygdala-hippocampus couple, is believed to be involved in the encoding and retrieval of emotionally charged memories associated with pain perception (Neugebauer et al. 2009). Their strong anatomical interconnections and shared functions (McDonald and Mott 2017) may readily explain why they are clustered together. The low connectivity of the limbic module with other nodes in the present analysis is, however, surprising. Some functional independence of these regions was also detected in iEEG data, which showed no significant spectral coherence between the amygdala and the posterior insula (Bastuji et al. 2018). Nociceptive inputs reach the amygdala through a low speed tract (the spino-parabrachial-amygdalar pathways; Bernard and Besson 1990) leading to responses with a different temporal shape than other cortical regions (see Fig. 4 in Bastuji et al. 2018). Such desynchronized mode of response has also been shown for hippocampal iEEG responses and might have contributed to the desynchronization of limbic structures with the rest of the network in our experiment. However, the functional interplay between the hippocampus and the PCC/precuneus, and between the amygdala and the aI/orbito-frontal nodes are well documented (Neugebauer et al. 2009), and hence, this lack of interaction remains enigmatic. It may be hypothesized that under experimental conditions with low emotional impact as was the case here, stimulus processing in limbic areas may remain very limited and largely uncorrelated from that in sensory and cognitive networks.

In line with this, the connectivity with limbic areas was higher in the audio condition, which was based on verbal comments with significant emotional impact (Fauchon et al. 2019). The connectivity of number of nodes was stimulus dependent. Intramodular connectivity was higher in response to pain than to audio stimuli, especially between nodes of the sensorimotor network, and is in favor of a specific network structure of the brain regions involved in pain, associated with a higher small worldness. One broader interpretation of these results is that specialized subsystems (modules) enhance their internal communications for integrating the multiple processes associated with pain experience (as also shown in Zheng et al. 2019), while the between subsystems communication and synchronization is dedicated to specialized structures represented by dynamical and connector hubs.

### The Essential Links: Modular Connectors

The main hubs detected in our data were located in posterior parietal, dorsolateral prefrontal, and anterior insular cortices. Modular connector hubs provide connection of different modules and are thought to serve critical roles in coordinating network integrity (van den Heuvel and Sporns 2013); accordingly, such connectors were primarily concentrated in association with cortices and regions that support multiple cognitive processes, as has been described in previous literature (Cole et al. 2013; Bertolero et al. 2015; Liang et al. 2016). Of note, the anterior insula also lied on the most direct route between any two-node combinations in the network and was accordingly identified as a “dynamical hub.” Nodes with such characteristics (defined as high “betweenness centrality,” see Methods) play a key role in network organization by virtue of their control over information passing between other nodes (Joyce et al. 2010), but are also, logically, loci of vulnerability in brain disorders (van den Heuvel and Sporns 2013).

The aI is an area of functional convergence, with extensive reciprocal connections to and from other areas (Craig 2009) and ensuring rapid integration of sensory and limbic nociceptive input (Bastuji et al. 2018). Neuroimaging studies examining the directional influences exerted by the aI suggested that this area may play a causal role in switching between the DMN and CE network (Sridharan et al. 2008; Bressler and Menon 2010). However, its activity largely exceeds the modulation of executive functions and also influences affective systems, autonomic functions, and general cognitive processes (Craig 2009; Nieuwenhuys 2012). The high “centrality” of the aI, reflecting its control over passing information, is consistent with a role in orchestrating potentially necessary changes in behavioral response (Blair 2016). The continuous dialogue between the four modules through the anterior insula and the highly interconnected fronto-parietal modules could contribute to link external and internal sensory worlds. While necessarily speculative, this interpretation would be consistent with a model of continuous intrinsic fluctuations among densely collaborating regions that has been described as a “dynamic pain connectome” (Kucyi and Davis 2015).

## Limitations and Conclusion

The standpoint of this study was a restricted network derived from iEEG recordings, focused on 34 areas responding with known temporal dynamics to nociceptive input; therefore, generalization to other sensory modalities may not be straightforward. While our graph analysis was based on the functional connectivity between these brain regions, an association does not necessarily correspond to a direct connection or involve causal relationships. Thus, it is not possible to infer about the directionality and/or causality of connections, except the fact that they corresponded to components whose response latency is known from iEEG recordings. To further isolate specific correlates of physical pain, future work should include the use of different intensities, including below nociceptive thresholds (Horing et al. 2019). Also, the experiments reported here were conducted in very reassuring contexts where emotional drive from pain-related fear or anxiety was purposely attenuated, thus probably minimizing the role of limbic regions in the overall network. This is doubtless the case of a vast majority of experimental pain studies and limits the ecological validity of the results in these “overprotective” contexts. Notwithstanding such limitations, the modular organization reported here appeared relevant to shape the dynamic patterns of responses to pain stimuli determined at short time scale using iEEG and are, therefore, consistent with the brain connectivity properties being reproduced at short and long time scales (Nagy et al. 2017). The results are consistent with a model of nociceptive integration whereby the conscious experience of pain emerges from the dynamic cooperation, segregation, and integration of multiple functional subsystems. It provides a picture of the “brain in pain” as a functional wiring diagram and may hopefully help to understand brain network reorganizations occurring in patients with chronic pain.

## Notes

The data are not publicly available due to third party restriction and subject privacy issues of the institution. The data that support the findings of this study are available upon reasonable request to the corresponding author. *Conflict of Interest:* The authors declare no competing interests.

## Funding

Labex CORTEX (ANR-11-LABX-0042, ANR-11-IDEX-0007); Academic Research Community (ARC-2) of Region Rhône-Alpes, France (to C.F.); Institut UPSA de la douleur, the APICIL foundation, and Novartis France.

## Code and Software Accessibility

NeuroPycon is freely available for download via github (https://github.com/neuropycon), the graphpype package is documented online https://neuropycon.github.io/graphpype/index.html.

Computations using radatools to analyze complex networks is also freely distributed (http://deim.urv.cat/~sergio.gomez/radatools.php).

## Supplementary Material

Supplemental_material_tgaa088
