# Supplementary material for: The Modular Organization of Pain Brain Networks: An fMRI Graph Analysis Informed by Intracranial EEG
Source: Cereb Cortex Commun. 2020 Nov 25;1(1):tgaa088. doi: 10.1093/texcom/tgaa088 (PMC8152828; doi:10.1093/texcom/tgaa088)
Supplement: Supplemental_material_tgaa088 [file supplemental_material_tgaa088.docx]

**Glossary of graph terms** (also see Fornito et al. 2016)

**Graphs**

A graph is a mathematical object, composed of a set of nodes, and a set of links (or “edges”) between pairs of the nodes. The nodes represent (sub)cortical brain regions, and the edges represent functional connections.

**Path length**

A path is a series of edges connecting two nodes in a graph. The path length is the number of edges in a path. Out of all possible paths between two nodes, the shortest path length corresponds to the path made up of the fewest edges. Path length is inversely related to the efficiency of information transfer in a network.

**Clustering**

A high clustering coefficient means that the nearest neighbors of a given node have a high probability to be connected with each other to form the topological motif of a triangle. Small-world networks have higher clustering, but approximately equivalent path length, compared to a random network.

**Small world networks**

A class of networks that show high clustering coefficient, much like a lattice, but with a short characteristic path length, much like a random graph. The human brain is contemplated as a small world network.

**Centrality**

The influence or importance of a node or edge in network function. This influence is defined in relation to the connection topology of the node or edge.

**Degree centrality**

The number of edges or links that connect a node to the rest of the network.

**Betweenness centrality**

A measure of topological centrality based on the fraction of shortest paths that pass through a given node. A node with high betweenness is described as a mediator or broker of network integration.

**Modularity**

The division of the nodes of a network into strongly interconnected subsets called modules (or communities). Networks can be decomposed into modules. By definition, there is higher connectivity between nodes belonging to the same module than between nodes that belong to different modules.

**Module**

A subset of nodes that are strongly interconnected with each other and sparsely interconnected with nodes in other modules. Once the modular structure is determined, the density of inter- and intra- modular connections of individual nodes is assessed using respectively the ***participation coefficient*** (PC: density of links that connect a node between modules) and the ***within-module degree*** (WMD: number of links that connect a node within a module).

**Hubs**

An important or topologically central node in a network. Different measures of centrality have been used to define hubs and to distinguish between three different types of hubs.

Nodes with a relatively large number of connections, i.e., high degree, are described as **static hubs**, whereas nodes mediating a high proportion of information flow, i.e., high betweenness centrality, are described as **dynamical hubs**.

**Connector hubs**

In a modular system, some nodes may have a special role to play in mediating the relatively sparse connections between different modules. In brain networks, cortical regions with greater inter-modular connectivity, ie, high Participation coefficient, are called **connector hubs**.

**Reference**

Fornito A, Zalesky A, Bullmore ET (Eds.). 2016. Chapter 1 - An Introduction to Brain Networks. In: Fundamentals of Brain Network Analysis. San Diego: Academic Press. p. 1–35.

**Supplemental Table 1.** Global graph metrics associated with pain (group 1 & 2) and audio (group 1).

|  | **Assortativity** | **Global efficiency** | **Clustering coefficient** | **Modularity** | **Small-worldness coefficient** |
| --- | --- | --- | --- | --- | --- |
| **Group 1, Pain** | 0.55 | 0.59 | 0.75 | 0.40 | 2.16 |
| **Group 2, Pain** | 0.54 | 0.57 | 0.74 | 0.37 | 2.17 |
| **Group 1, Audio** | 0.56 | 0.5 | 0.68 | 0.35 | 1.75 |

**Supplemental Table 2.** Nodal graph metrics associated with the brain modular partition during pain in group 1. Clustering coefficient, degree, participation coefficient, and the Z wiyhin-module degree are reported for left (L) and right (R) nodes: Parietal operculum (S2), Posterior insula (pI), Anterior insula (aI), Frontal operculum (Foper), Primary somatosensory cortex (S1), Supplementary motor area (SMA), Perigenual ant. cingulate cortex (pACC), Anterior cingulate cortex (ACC), Mid- cingulate cortex (MCC), Dorsal post.cingulate cortex (dPCC), Ventral post. cingulate cortex (vPCC), Post. parietal cortex (PPC), Precuneus (Prec), Orbito frontal cortex (OFC), Middle frontal gyrus (DLPFC), Amygdala (Amyg), Hippocampus (Hip).

| **Module**  **Group 1** | **Node** | **Clustering coef.** | **Degree** | **Participation coef.** | **Z within-module degree** |
| --- | --- | --- | --- | --- | --- |
| **Sensory-motor (SM)** | Foper_L | 0.86 | 15.00 | 0.32 | 0.32 |
|  | Foper_R | 0.69 | 16.00 | 0.41 | 0.67 |
|  | aI_L | 0.65 | 17.00 | 0.51 | 2.00 |
|  | aI_R | 0.78 | 15.00 | 0.44 | 0.83 |
|  | pI_L | 0.95 | 13.00 | 0.14 | -0.37 |
|  | pI_R | 0.95 | 13.00 | 0.14 | -0.37 |
|  | MCC_L | 0.76 | 15.00 | 0.34 | 0.32 |
|  | MCC_R | 0.86 | 15.00 | 0.32 | 0.32 |
|  | S1_L | 0.76 | 13.00 | 0.26 | -0.37 |
|  | S1_R | 0.96 | 5.00 | 0.00 | 0.24 |
|  | S2_L | 0.86 | 14.00 | 0.13 | -1.51 |
|  | S2_R | 0.87 | 13.00 | 0.00 | -1.51 |
|  | SMA_L | 0.73 | 17.00 | 0.36 | 1.02 |
|  | SMA_R | 0.78 | 16.00 | 0.30 | 0.67 |
| **Medial fronto-parietal (med-FP)** | OFC_L | 0.75 | 8.00 | 0.00 | 0.77 |
|  | OFC_R | 0.75 | 8.00 | 0.00 | 0.77 |
|  | ACC_L | 0.47 | 6.00 | 0.61 | -0.91 |
|  | ACC_R | 0.47 | 6.00 | 0.61 | -0.91 |
|  | Prec_L | 0.79 | 8.00 | 0.00 | 0.77 |
|  | Prec_R | 0.86 | 7.00 | 0.00 | -0.07 |
|  | dPCC_L | 0.79 | 8.00 | 0.00 | 0.59 |
|  | dPCC_R | 0.60 | 5.00 | 0.32 | -1.94 |
|  | pACC_L | 0.54 | 8.00 | 0.38 | 0.59 |
|  | pACC_R | 0.60 | 5.00 | 0.40 | -1.94 |
|  | vPCC_L | 0.71 | 8.00 | 0.22 | 0.59 |
|  | vPCC_R | 0.68 | 8.00 | 0.22 | 0.59 |
| **Lateral fronto-parietal (lat-FP)** | DLPFC_L | 0.75 | 11.00 | 0.51 | 0.77 |
|  | DLPFC_R | 0.74 | 12.00 | 0.49 | 1.10 |
|  | PPC_L | 0.57 | 7.00 | 0.34 | -1.57 |
|  | PPC_R | 0.79 | 14.00 | 0.61 | 1.18 |
| **Limbic** | Amyg_L | 0.67 | 3.00 | 0.00 | 1.00 |
|  | Amyg_R | 1.00 | 2.00 | 0.00 | -1.00 |
|  | Hip_L | 1.00 | 2.00 | 0.00 | -1.00 |
|  | Hip_R | 0.67 | 3.00 | 0.00 | 1.00 |

**Supplemental Table 2.** Nodal graph metrics associated with the brain modular partition during pain in group 2. Clustering coefficient, degree, participation coefficient, and the Z wiyhin-module degree are reported for left (L) and right (R) nodes: Parietal operculum (S2), Posterior insula (pI), Anterior insula (aI), Frontal operculum (Foper), Primary somatosensory cortex (S1), Supplementary motor area (SMA), Perigenual ant. cingulate cortex (pACC), Anterior cingulate cortex (ACC), Mid- cingulate cortex (MCC), Dorsal post.cingulate cortex (dPCC), Ventral post. cingulate cortex (vPCC), Post. parietal cortex (PPC), Precuneus (Prec), Orbito frontal cortex (OFC), Middle frontal gyrus (DLPFC), Amygdala (Amyg), Hippocampus (Hip).

| **Module**  **Group 2** | **Node** | **Clustering coef.** | **Degree** | **Participation coef.** | **Z within-module degree** |
| --- | --- | --- | --- | --- | --- |
| **Sensory-motor (SM)** | Foper_L | 0.83 | 14.00 | 0.26 | 0.79 |
|  | Foper_R | 0.67 | 14.00 | 0.34 | 1.20 |
|  | aI_L | 0.63 | 16.00 | 0.53 | 2.02 |
|  | aI_R | 0.74 | 14.00 | 0.51 | 0.83 |
|  | pI_L | 0.90 | 10.00 | 0.14 | -0.86 |
|  | pI_R | 0.90 | 11.00 | 0.14 | -0.86 |
|  | MCC_L | 0.69 | 15.00 | 0.27 | 0.79 |
|  | MCC_R | 0.79 | 14.00 | 0.32 | 0.38 |
|  | S1_L | 0.76 | 13.00 | 0.23 | -0.31 |
|  | S1_R | 0.91 | 9.00 | 0.16 | 0.34 |
|  | S2_L | 0.84 | 14.00 | 0.00 | -0.45 |
|  | S2_R | 0.88 | 13.00 | 0.00 | -1.27 |
|  | SMA_L | 0.90 | 14.00 | 0.22 | 1.27 |
|  | SMA_R | 0.80 | 15.00 | 0.32 | 0.45 |
| **Medial fronto-parietal (med-FP)** | OFC_L | 0.77 | 9.00 | 0.00 | 0.40 |
|  | OFC_R | 0.77 | 9.00 | 0.00 | 0.40 |
|  | ACC_L | 0.48 | 7.00 | 0.57 | -0.91 |
|  | ACC_R | 0.50 | 8.00 | 0.63 | -0.91 |
|  | Prec_L | 0.74 | 10.00 | 0.18 | 0.29 |
|  | Prec_R | 0.83 | 10.00 | 0.32 | 0.29 |
|  | dPCC_L | 0.74 | 7.00 | 0.18 | 0.29 |
|  | dPCC_R | 0.59 | 5.00 | 0.26 | 2.37 |
|  | pACC_L | 0.53 | 10.00 | 0.00 | 0.29 |
|  | pACC_R | 0.63 | 6.00 | 0.00 | 0.98 |
|  | vPCC_L | 0.75 | 8.00 | 0.22 | -0.40 |
|  | vPCC_R | 0.67 | 7.00 | 0.22 | -0.40 |
| **Lateral fronto-parietal (lat-FP)** | DLPFC_L | 0.71 | 11.00 | 0.49 | 1.67 |
|  | DLPFC_R | 0.77 | 9.00 | 0.57 | 1.03 |
|  | PPC_L | 0.50 | 8.00 | 0.59 | -1.00 |
|  | PPC_R | 0.76 | 14.00 | 0.67 | 1.20 |
| **Limbic** | Amyg_L | 0.67 | 3.00 | 0.00 | 1.00 |
|  | Amyg_R | 1.00 | 2.00 | 0.00 | -1.00 |
|  | Hip_L | 1.00 | 3.00 | 0.00 | -1.00 |
|  | Hip_R | 0.67 | 3.00 | 0.00 | 1.00 |
